# Editorial: Environmental toxicity in metabolism

**DOI:** 10.3389/ftox.2026.1925459

**Published:** 2026-08-21

**Authors:** Tracey Woodlief

**Affiliations:** Department of Pharmacology and Toxicology, East Carolina University, Greenville, NC, United States

**Keywords:** endocrine disruption, environmental toxicology, lipid metabolism, metabolic reprogramming, mitochondrial dysfunction, oxidative stress, Per- and polyfluoroalkyl substances, peroxisomal β-oxidation

## Introduction

Environmental chemical exposures are increasingly recognized as critical determinants of metabolic disease risk. Among these, per- and polyfluoroalkyl substances (PFAS) are of particular concern due to their environmental persistence, bioaccumulation, and strong mechanistic links to dysregulated lipid metabolism and hepatic dysfunction. Emerging evidence supports the concept that PFAS act not as inert contaminants, but as biologically active metabolic disruptors capable of reprogramming systemic energy homeostasis and cellular bioenergetics.

This Research Topic, *Environmental Toxicity in Metabolism*, integrates experimental, mechanistic, and systems-level studies that collectively support a paradigm in which environmental chemicals actively reshape metabolic networks. Recent PFAS-focused work highlights sex-dependent lipid metabolic disruption and hepatic steatosis mechanisms involving PPAR signaling and bile acid metabolism regulation (Hari et al., 2024), reinforcing PFAS as central drivers of metabolic imbalance.

### PFAS as lipid mimics and nuclear receptor modulators

A central mechanistic theme emerging across this Research Topic is the structural similarity of PFAS to endogenous fatty acids, enabling interactions with lipid-sensing nuclear receptors such as peroxisome proliferator-activated receptors (PPARs). Through these interactions, PFAS influence transcriptional programs governing fatty acid transport, β-oxidation, and lipid storage.

Mechanistic reviews demonstrate that PFAS exposure consistently disrupts lipid metabolic pathways and contributes to dyslipidemia and fatty liver disease through receptor-mediated signaling perturbations and altered lipid homeostasis (Cui et al., 2020). These findings are supported by more recent studies linking PFAS exposure to sex-dependent alterations in rate-limiting enzymes governing hepatic lipid flux (Hari et al., 2024).

### Mitochondrial dysfunction and bioenergetic stress

Mitochondrial impairment is a consistent mechanistic outcome of PFAS exposure. PFAS reduce oxidative phosphorylation efficiency, increase reactive oxygen species (ROS), and disrupt electron transport chain function, collectively producing a state of bioenergetic stress.

Recent experimental evidence demonstrates chain-length–dependent mitochondrial toxicity among perfluoroalkyl acids, linking PFAS molecular structure directly to mitochondrial dysfunction severity and metabolic failure (Kam et al.). These effects converge on impaired fatty acid oxidation and accumulation of incomplete lipid intermediates, establishing mitochondrial dysfunction as a central node in PFAS-induced metabolic disease.

### Peroxisomal compensation and redox imbalance

PFAS exposure induces compensatory activation of peroxisomal β-oxidation pathways, including upregulation of acyl-CoA oxidase 1 (ACOX1). While initially adaptive, this response generates hydrogen peroxide as a metabolic byproduct, increasing oxidative stress burden.

The resulting mitochondrial–peroxisomal imbalance produces a redox disequilibrium that amplifies lipid peroxidation, stress signaling pathways, and hepatocellular injury. This metabolic inefficiency represents a defining feature of PFAS-driven bioenergetic disruption.

### Hepatic metabolic reprogramming as a central toxicity axis

The liver is consistently identified as a primary target organ for PFAS toxicity. Across experimental systems, PFAS exposure is associated with hepatomegaly, altered lipid distribution, and accumulation of triglycerides and bioactive lipid intermediates.

Mechanistically, PFAS alter hepatic fatty acid uptake, mitochondrial β-oxidation, and lipid export pathways, producing coordinated metabolic reprogramming. Importantly, recent studies demonstrate that these hepatic effects are often sex-dependent and associated with disruptions in gluconeogenesis, bile acid metabolism, and lipid synthesis pathways (Hari et al., 2024).

### Endocrine disruption and systemic metabolic effects

Beyond direct metabolic effects, PFAS disrupt endocrine signaling networks including thyroid hormone regulation, glucocorticoid signaling, and insulin sensitivity. These hormonal disruptions interact with intracellular metabolic pathways to produce multi-system metabolic dysregulation.

The convergence of endocrine disruption and mitochondrial stress supports the concept that PFAS function as systems-level metabolic reprogrammers rather than isolated pathway toxicants, affecting inter-organ communication and whole-body energy balance.

## Mixture toxicology and real-world exposure complexity

Human exposure to PFAS occurs as complex mixtures rather than single compounds, including legacy and emerging PFAS with varying chain lengths and biological persistence. Recent studies demonstrate that mixture exposures produce non-linear and sometimes synergistic metabolic effects, including lipid dysregulation and oxidative stress amplification.

These findings align with broader PFAS toxicology literature demonstrating that biological outcomes depend on chemical structure, mixture composition, and physiological context, reinforcing the need for mixture-aware risk assessment frameworks (Kang et al., 2026).

## Emerging mechanisms in PFAS-driven metabolic toxicity

Recent work expands PFAS toxicology into additional mechanistic domains, including ferroptosis, lipid peroxidation pathways, and regulated cell death processes driven by oxidative lipid damage.

Transcriptomic and systems-level studies further demonstrate PFAS-induced tissue-specific metabolic reprogramming and broad molecular remodeling across multiple organs, reinforcing their classification as multi-system metabolic disruptors (Wang et al., 2025).

### Toward integrated environmental metabolic toxicology

Collectively, the studies included in this Research Topic span mechanistic, mitochondrial, systems biology, and mixture toxicology frameworks, supporting an integrated model in which environmental exposures actively reshape metabolic biology.

Future priorities include defining exposure thresholds for early metabolic reprogramming, resolving tissue-specific PFAS partitioning, quantifying mitochondrial–peroxisomal flux dynamics, and integrating mixture toxicology into regulatory frameworks. Importantly, linking molecular signatures of PFAS exposure to epidemiological outcomes will be essential for translating mechanistic insights into public health relevance.

## Conclusion

The contributions within this Research Topic support a paradigm shift in which PFAS are recognized as active regulators of metabolic biology rather than passive environmental contaminants. Through coordinated disruption of lipid sensing pathways, mitochondrial bioenergetics, peroxisomal oxidation, endocrine signaling, and immune-metabolic networks, PFAS fundamentally reprogram energy metabolism across biological systems.

By centering PFAS within environmental metabolic toxicology, this Research Topic advances a mechanistic framework connecting persistent environmental exposure to the global burden of metabolic disease. This integrated perspective provides a foundation for improved toxicological modeling, refined risk assessment, and ultimately more effective strategies to mitigate environmentally driven metabolic dysfunction.
